# Understanding how adolescents engage in mindfulness-based intervention: a mixed methods study of engagement processes

**DOI:** 10.1007/s12144-026-09542-3

**Published:** 2026-05-27

**Authors:** Megan J. Moran, Talia Thompson, Michelle Harmon, Addie Rzonca, Rachel G. Lucas-Thompson, Melissa DeJonkheere, Timothy C. Guetterman, Lauren B. Shomaker

**Affiliations:** 1 Department of Human Development and Family Studies, College of Health and Human Sciences, Colorado State University, Fort Collins, United States; 2 Department of Pediatrics, Section of Endocrinology, University of Colorado Anschutz Medical Campus and Children’s Hospital Colorado, Aurora, United States; 3 University of Colorado Anschutz Medical Campus/Children’s Hospital Colorado, Aurora, United States; 4 University of Michigan, Ann Arbor, United States

**Keywords:** Mindfulness-based intervention, Qualitative, Mixed methods, Engagement, Adolescent

## Abstract

Mindfulness-based intervention (MBI) improves mental health outcomes in adolescents; however, effects tend to be small, and findings have been inconsistent. Engagement (i.e., productive involvement) has been positively associated with outcomes in other types of mental health interventions, suggesting it may play a role in MBI. However, it is unclear how engagement functions in the context of MBI for adolescents. The purpose of this mixed methods study was to examine adolescent engagement in MBI, including identifying. reasons they do or do not engage. In *n* = 73 adolescents who received a community-delivered MBI, we described self-reported engagement and tested differences between engagement dimensions. Based on quantitative scores, we purposively sampled *n =* 25 adolescents for interviews and used reflexive thematic analysis to develop themes that reflected adolescents’ perspectives on engaging in MBI. Through merging integration, we examined patterns of thematic results across engagement levels. Engagement scores were high, and cognitive-behavioral engagement was significantly higher than affective engagement. Qualitative and mixed methods analyses resulted in four themes that showed variation by engagement level. Adolescents’ perspectives on engagement in MBI aligned somewhat with prior conceptualizations of engagement, and highlighted distinctive characteristics of engagement in the MBI context. There are opportunities before, during, and outside of MBI sessions to support adolescent engagement, which may increase efficacy of MBI for promoting mental health in this age group.

Mental health problems among U.S. adolescents increased between 2013 and 2023, with 40% of high school students reporting persistent feelings of sadness and hopelessness in 2023 (Centers for Disease Control and Prevention, 2024). It is critical to invest in prevention efforts targeted at adolescents to strengthen factors that protect against mental health problems. Mindfulness-based intervention (MBI) has been associated with positive outcomes in adolescents, including improvements in emotion regulation, a key protective factor in mental health, as well as reductions in symptoms of psychopathology (e.g., depression, anxiety) and subjective distress (Dunning et al., 2019; Fulambarkar et al., 2023; Klingbeil et al., 2017). However, MBI findings related to key mental health outcomes have been inconsistent (Fulambarkar et al., 2023), suggesting our understanding of the mechanisms underlying MBI remains insufficient. One potential mechanism is engagement, or the processes that indicate productive involvement with an activity (Ben-Eliyahu et al., 2018). Engagement may play a role in MBI, but it is unclear whether engagement carries the same meaning or functions similarly in the context of MBI for adolescents as it does in school and out-of-school learning environments. Given the well-established role of engagement in learning processes (Wong et al., 2024), the potential of engagement to act as an underlying mechanism of MBI efficacy, and the limited research on engagement within MBI, mixed methods investigation of this construct that prioritizes the voices of adolescents who demonstrate varying levels of engagement is critical to inform the refinement of MBI for maximal impact.

Converging evidence supports the potential of mindfulness and MBI to improve adolescent mental health outcomes. Dispositional mindfulness (i.e., present-centered, nonjudgmental attention) has been associated with fewer mental health problems in adolescents (Cortazar & Calvete, 2019; Johnson & Wade, 2019; Kabat-Zinn, 1990; Royuela-Colomer et al., 2021). Moreover, MBI, which involves teaching participants what mindfulness is and how to practice, increases dispositional mindfulness in adolescents and reduces depression and anxiety symptoms (Dunning et al., 2019; Klingbeil et al., 2017; Zhang et al., 2021). Despite a signal that MBI may be a promising prevention strategy for mental health problems, meta-analytic effects tend to be small (Fulambarkar et al., 2023; Klingbeil et al., 2017). Small effect sizes can indicate that an intervention works for some, but not for others (Kraft, 2020), and indeed, evidence from recent large-scale randomized controlled trials suggests that MBI does not produce the desired outcomes in all adolescents (Montero-Marin et al., 2022). Patterns of small effects indicate the need for deeper understanding of processes occurring during and between intervention sessions when delivering MBI to this age group.

To better understand these variable effects, it is important to examine underlying intervention processes, such as engagement. Engagement has been extensively studied in learning contexts. It is recognized as critical to school-based learning and has been positively associated with outcomes in other types mental and behavioral health interventions, including in adolescents (Bijkerk et al., 2022; Marker et al., 2013). Most of the work on engagement acknowledges its multidimensionality and posits two to three dimensions (Ben-Eliyahu et al., 2018; Fredricks, 2011). We utilize a two-dimension model of engagement comprised of cognitive-behavioral and affective components (Ben-Eliyahu et al., 2018; Bijkerk et al., 2022; Fredricks, 2011; Holdsworth et al., 2014) (Fig. 1). Cognitive-behavioral engagement reflects effortful participation (Ben-Eliyahu et al., 2018) that is dedicated to both cognitive tasks associated with the activity (e.g., thinking, paying attention, gaining understanding) as well as behavioral tasks, which are highly variable from one specific activity to next, but broadly relate to taking initiative. In a group learning context, behavioral engagement includes behaviors such as commenting or sharing, responding to a question from a teacher/facilitator, or nodding (Ben-Eliyahu et al., 2018). Affective engagement reflects the extent of positive affective reactions to an activity, such as interest or enjoyment, as well as calm or relaxation. Importantly, both cognitive-behavioral and affective dimensions of engagement reflect a dynamic process between a learner or participant and the context and, thus, is highly context-dependent (Ben-Eliyahu et al., 2018; Fredricks et al., 2004; Lam et al., 2012; Pöysä et al., 2018). As MBI programs involve learning, about what mindfulness is and how to practice it, we anticipate that engagement in MBI overlaps to some extent with engagement in other learning contexts. However, mindfulness practice may challenge conventional assumptions embedded in engagement frameworks. For example, cognitive-behavioral engagement typically emphasizes effortful participation, active contribution, and goal-directed behavior (Ben-Eliyahu et al., 2018; Fredricks, 2011), whereas mindfulness emphasizes non-striving, present-moment awareness, and nonjudgmental acceptance (Kabat-Zinn, 1990; Shapiro et al., 2018). As such, higher observable or effortful engagement may not necessarily reflect more effective mindfulness practice, and some forms of engagement (e.g., quiet, internal attention) may be less visible or not captured by traditional measures. Thus, it is not known the degree to which prior conceptualizations of engagement are relevant or appropriate in the MBI context.

**Fig. 1:**
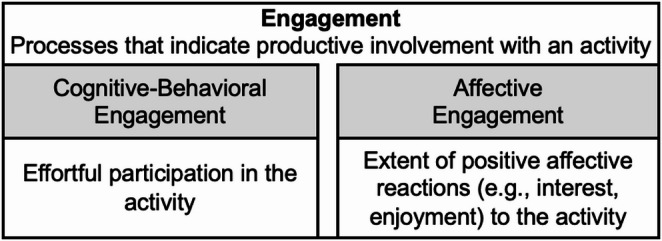
Multidimensional Engagement

Engagement predicts better outcomes in learning contexts and psychotherapeutic interventions (Bijkerk et al., 2022; Wong et al., 2024; Yew et al., 2021). For example, in a single-arm study of children receiving therapy for anxiety, therapeutic alliance (related to engagement) was associated with significant reductions in anxiety (Marker et al., 2013), and in a single-arm study of adults with depression receiving cognitive-behavioral therapy, willingness to complete therapy homework (a form of cognitive-behavioral engagement) was associated with greater reductions in depression symptoms (Neimeyer et al., 2008). Further, one study of MBI in adults found that engagement predicted increases in some facets of mindfulness from baseline to post-intervention (Banerjee et al., 2018). There have been minimal examinations of engagement during MBI in adolescents, leaving a critical gap in our understanding of this process, despite its potential implications for intervention efficacy for this age group (Wong et al., 2024).

Better understanding of engagement in adolescent MBI is needed because engagement may be particularly important when delivering MBI to adolescents, relative to adults. Although some MBIs are designed to make mindfulness more accessible and concrete, mindfulness is nonconceptual and paradoxical, which may make it challenging for adolescents to put into practice in the ways that, theoretically, afford the most benefit (Shapiro et al., 2018). Moreover, because the neural circuitry that supports the capacity for mindfulness is still developing in adolescents (Tang et al.,^ 2015^), mindfulness practice may be more difficult for adolescents than adults. As a result, adolescents likely need more and/or different types of support to fully engage with MBI. Importantly, engagement may be a valuable intervention process to target because it is presumed to be malleable, dynamic, and responsive to context (e.g., learning environment, peers, etc.) (Fredricks et al., 2004; Furlong & Christenson, 2008). Thus, there is a need to identify specific, subjective facilitators and barriers to engagement experienced within this age group.

## Current study

This mixed methods study aimed to support the continued refinement of MBI for the prevention of mental health challenges in adolescents by describing levels of engagement in a group-based, multi-session, manualized MBI, as well as the meaning of engagement to adolescents, including identifying the reasons that they do or do not engage. We sought to address these aims through characterizing quantitatively measured engagement and by interviewing adolescents who represented a wide range of levels of engagement, as measured by quantitative surveys, about their experience in an MBI. In analyzing interview responses, we inductively developed themes that reflected adolescents’ own understandings of and perspectives on their experience in an MBI, as well as identified whether adolescents’ descriptions of engagement in MBI mapped onto well-established conceptualizations of engagement.

## Materials and methods

### Study design

**Fig. 2:**
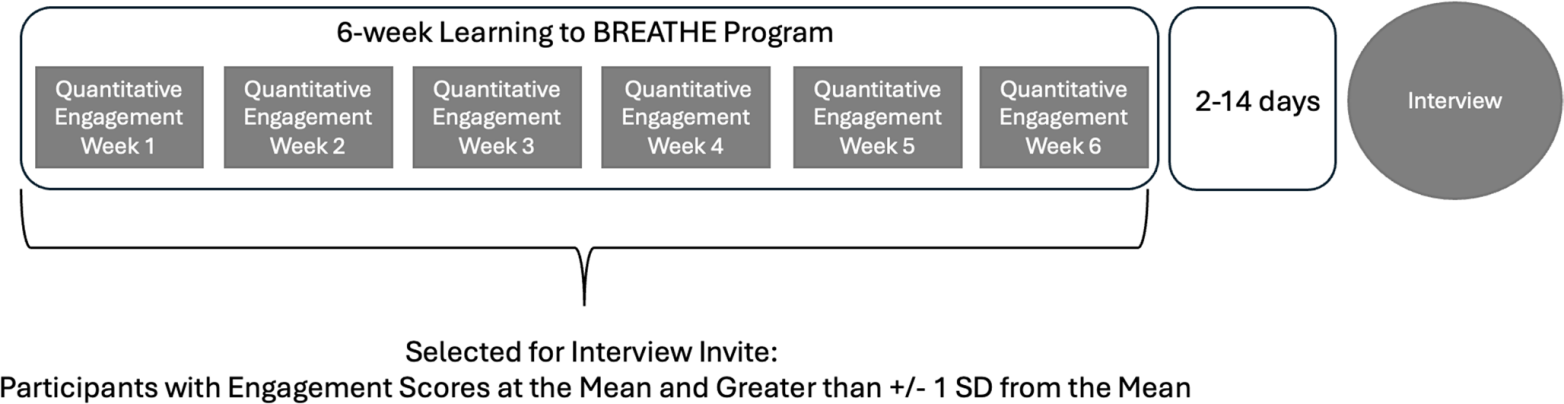
Explanatory Sequential Mixed Methods Intervention Design

The present mixed methods study is part of a larger single-arm pilot examination of engagement and mechanistic outcomes of MBI in non-clinical adolescents. We utilized an explanatory sequential mixed methods design embedded in an intervention study (Creswell & Plano Clark, 2017), whereby quantitative measures of engagement, collected from the full sample at six weekly intervals during the MBI, informed the selection of a subsample of participants for post-intervention interviews (Fig. 2). All study procedures were approved by the (masked for review) Institutional Review Board.

### Participants

Participants were *n* = 73 community adolescents, of which *n* = 25 completed post-intervention interviews. Participants were recruited through flyers, emails to relevant listservs, referrals from youth services organizations, and social media ads. Participants who were 18 years of age provided consent; those under age 18 years provided assent and a parent or guardian provided consent. During the consent procedures, participants were informed that some but not all of the adolescents receiving the intervention would be asked to do an interview after the intervention ended. Adolescents received $20 for completing the majority (≥ 5 out of 6) of weekly quantitative surveys or $5 if they completed fewer than 5 surveys. Interview participants were provided with a $20 gift card for their time.

### Intervention

Enrolled adolescents received Learning to BREATHE (L2B) (Broderick, 2021), an evidence-based manualized MBI that was designed to be developmentally suited for adolescents. The program was delivered to eight consecutive cohorts between June 2023 and July 2024. Cohort size ranged from 6 to 12 adolescents (Median = 8.5) and included adolescents spanning the full age range of the sample (12-18y) resulting in mixed-age groups. Adolescents attended 1.5-hour group sessions once a week for six weeks, in-person at a community location (e.g., private meeting rooms at local community recreational centers). Every L2B session involves a combination of several components: (1) didactic content on what mindfulness is and how to practice it in daily life, (2) experiential activities to deepen adolescents’ understanding of mindfulness, commonly involving facilitated group discussion, and (3) relatively brief (3–12 min) guided meditations (Broderick, 2021). Participants are provided with home practice suggestions, including links to guided audio practices to do independently. Sessions were led by two trained facilitators, including the first author, who is a certified master trainer of L2B.

#### Quantitative phase

##### Data collection

At the end of each of the six weekly L2B sessions, participants completed paper self-report measures of engagement. To assess *cognitive-behavioral engagement*, adolescents completed the 7-item behavioral engagement subscale and the 4-item cognitive engagement subscale of the Activity Engagement Scale (AES), an instrument that has been used reliably to assess adolescent engagement in out-of-school contexts (Ben-Eliyahu et al., 2018). Items were modified to refer specifically to L2B (e.g., “Today in L2B, I contributed to the discussion”). On a scale of 1 (*not at all true*) to 5 (*very true*), adolescents rated the extent to which statements applied to them. Item responses were averaged at each week (Week 1: α = 0.82; Week 6: α = 0.84). To assess *affective engagement*, adolescents completed the 5-item affective engagement subscale from the AES (e.g., “In L2B, I felt frustrated or annoyed”; “In L2B, I felt happy or excited”). Item responses were averaged at each week (Week 1 α = 0.70; Week 6 α = 0.70).

For each type of engagement, we calculated each participant’s average score across the six weeks of the program. We then calculated the overall mean engagement score across participants. To identify adolescents to invite for interviews, each participant’s average engagement score was compared to this overall mean to determine whether their engagement score was above or below the sample average. The process was cumulative across cohorts, such that the overall mean was calculated using data from participants in that cohort and all previous cohorts.

##### Data analysis 

We calculated descriptive statistics using R (R Core Team, 2022). We also examined bivariate correlations between cognitive-behavioral and affective engagement and used paired-samples t-tests to test differences between scores on the two dimensions.

#### Qualitative phase

##### Data collection

We used connecting integration for interview sampling, whereby quantitative engagement scores were used to select participants for the interview phase. At the outset of the study, we set a target sample size for interviews based on the concept of information power, which considers the nature, complexity, and scope of the phenomenon under investigation, purposive sample parameters, and the analytic approach, among other elements (Malterud et al., 2016; Morse, 2015). We used purposive, maximum variation sampling to support the collection of richer, more variable qualitative data and, as a result, hoped to gain a deeper understanding of the phenomenon under investigation (Creswell, & Plano Clark, 2017). For each type of engagement, we first identified a pool of participants representing three levels of engagement: (1) average engagement (participants with scores closest to the mean), (2) low engagement (scores > 1 SD below the mean), and (3) high engagement (scores > 1 SD above the mean). We aimed to include approximately two participants per category when possible. From this pool, we randomly selected 3–5 participants at a time (depending on cohort size) to invite for interviews.

Qualitative data were collected through semi-structured interviews conducted between August 2023 and August 2024. Adolescents were interviewed by trained undergraduate students using Microsoft Teams. Interviewers did not have personal experience with the intervention and were not connected to intervention delivery. Interviewers explicitly invited honest feedback of the intervention, emphasized confidentiality of all interview data, and their own independence from intervention facilitators. Interviewers transcribed audio recorded interviews using Otter.ai software, de-identified them, and reviewed and compared transcripts to audio recordings for accuracy. On average, interviews were 26.2 min (SD = 6.1, Range = 15–41 min). Transcripts were uploaded into qualitative analytic software ATLAS.ti Mac Version 25 (*ATLAS.ti Scientific Software Development GmbH*, 2022) for organization and analysis.

##### Data analysis

We analyzed qualitative data within a contextualist interpretive framework with a pragmatic emphasis (Braun & Clarke, 2021; Creswell & Poth, 2017). We embraced the contextualist stance that multiple accounts of reality, each shaped by context and individual experience, are possible, as well as the role of the researcher as an active participant in the creation of contextually-situated and partial knowledge (Braun & Clarke, 2021). At the same time, we applied a pragmatic interpretive lens, emphasizing the role for both quantitative and qualitative research tools, and maintaining a focus on solutions to real world problems (Creswell & Poth, 2017) – namely, the need to better understand what it means to be engaged as an adolescent in MBI and how to support that engagement to improve adolescent mental health.

We followed Braun and Clarke’s (2006) six-stage approach to reflexive thematic analysis. The first author (##) was a primary coder, along with one other researcher, ##. The two coders developed the codebook collaboratively. ## coded all transcripts, and 80% of transcripts were co-coded for consensus. Our orientation to coding was primarily inductive, but included a hybrid of semantic codes to capture participants’ explicitly expressed meaning, as well as codes that reflected more latent constructs from the engagement and mindfulness literatures (Braun & Clarke, 2021; Fereday & Muir-Cochrane, 2006; Proudfoot, 2023). We also included a set of deductive codes related to how engagement has predominantly been defined in educational psychology, the field in which it has been most extensively studied (Ben-Eliyahu et al., 2018; Fredricks & McColskey, 2012). We applied these codes when appropriate, but continuously interrogated the fit between these codes and adolescents’ interview responses, and worked to practice reflexivity and protect against positivist assumptions (Braun & Clarke, 2021). Methodological rigor was supported through the first author’s prolonged engagement with the study participants, reflexive journaling by coders, the use of analytic memos to capture analytic choices, meetings to discuss coding differences, and multiple rounds of dialogue with the broader research team (Felner & Henderson, 2022; Morse,^ 2015^; Olmos-Vega et al., 2022).

#### Mixed methods analysis

First, we examined alignment of engagement concepts with data collection instruments to prepare for integrative analysis. Table 1 highlights the alignment between quantitative and qualitative data collection methods. After quantitative and qualitative data were analyzed separately, we used a joint display to merge quantitative and qualitative results through examining themes by level of quantitative engagement.

**Table 1:** Mixed Methods Data Collection

	Quantitative Survey Instruments	Representative Interview Questions
Cognitive-behavioral engagement	Activity Engagement Scale – Cognitive and Behavioral Engagement subscales	What helped you learn about mindfulness?What was hard about changing your habits to practice more mindfulness?Were you able to change any habits? What helped you do that?How much we participate in a group depends on a lot of things, and people have good reasons for how much or little they participate. On a scale of 1 to 5, how active of a participant do you think you were in this program? 1 would be that you didn’t participate a whole lot, and 5 would be you were really involved and actively participating. Why do you think you were a [insert number they shared]?
Affective engagement	Activity Engagement Scale- Affective Engagement subscale	(Interviewer share screen and show list of emotion words). Take a minute to look over this list of emotion words. Which of these words – you can choose a few! – fits best with how you usually felt when you were at L2B? Or is there a better emotion word that’s not on this list?What were some of the reasons that you usually felt [insert emotion they shared] in L2B? Do you think feeling [insert emotion they shared] affected the way you acted or participated in L2B?

## Results

Demographic information for the sample is provided in Table 2. Adolescents whose assigned sex at birth was female were overrepresented in the interview sample (χ²=3.87, *p*=.04), relative to the overall sample.

**Table 2:** Demographic Information about the Overall Sample and the Interviewed Subset

	Overall (*n* = 73)		Interviewed (*n* = 25)	
	*n*	%	*n*	%
**Assigned sex at birth**				
Female	39	53	18	72
Male	34	46	7	28
**Gender Identity**				
Girl	35	49	16	64
Boy	30	41	7	28
Non-binary, transgender, or gender fluid	4	5	2	8
Not provided	4	5	0	0
**Ethnicity**				
Hispanic	13	18	6	24
Non-Hispanic	58	78	19	76
Not reported	2	3	0	0
**Race**				
Native American	3	4	1	4
Asian	9	12	3	12
Black/African American	1	1	1	4
White	62	84	21	84
Another race	5	7	3	12
2 or more races	9	9	5	20
**Age**,** years** (M ± SD)	14.1 ± 2.0		14.0 ± 1.9	

### Quantitative results: descriptive statistics, association, and differences between engagement dimensions

In the overall sample, when averaged across timepoints, adolescents reported *M* = 4.30/5.00 (SD = 0.44) on cognitive-behavioral engagement and *M* = 3.77/5.00 (SD = 0.54) on affective engagement. Although only a few adolescents reported low levels of engagement, a large number of adolescents reported neutral engagement (3.0–4.0/5.0) (affective: 56% neutral; cognitive-behavioral: 21% neutral). In the interviewed subset, scores on composite measures of both dimensions were, descriptively, slightly higher (i.e., more positive valence) on average engagement than in the overall sample: *M*_cognitive−behavioral_=4.39, SD=0.57; *M*_affective_=4.07, SD=0.62.

Scores on the two dimensions were moderately, positively correlated in the overall sample (*r*=.57, *p*<.001), and more strongly in the interview sample (*r*=.76, *p*<.001).

To examine whether there were statistically significant differences between the two engagement dimensions, we conducted paired samples t-tests after confirming that the data were normally distributed using the Shapiro-Wilk test (overall sample: *p*=.26, interview sample: *p=*.16). In the overall sample, scores on cognitive-behavioral engagement were significantly higher than scores on affective engagement (*T* = 9.83, *df* = 72, *p*<.001), with a large effect (*d* = 1.15). Results were similar in the interview sample, in which cognitive-behavioral engagement was also significantly higher (*T =* 5.50, *df* = 24, *p*<.001), with large effect (*d* = 1.10).

### Qualitative results: interview themes

**Fig. 3:**
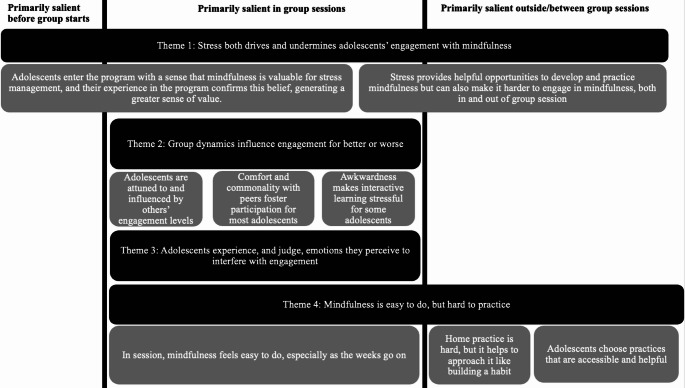
Summary of Thematic Results

Thematic analyses resulted in four themes and eight subthemes. In line with the pragmatic focus of our analyses, we then mapped these themes onto three phases of adolescents’ involvement with the MBI, as doing so could inform how to optimize the intervention for deeper engagement (Fig. 3). Themes were most salient at the following intervals: (1) before group sessions began, (2) during one or more of the six weekly 1.5-hour group sessions, and/or (3) outside or between group sessions. Some themes spanned multiple phases. All themes demonstrated conceptual connections to one or both dimensions of engagement (Ben-Eliyahu et al., 2018). Themes and subthemes are presented below, supported by illustrative quotes from adolescent participants. Participant identification number and gender, as well as their level of cognitive-behavioral engagement (CBE) and affective engagement (AE) are included along with quotes. Those adolescents identified as *low* reported engagement scores lower than the group mean; those identified as *high* reported engagement scores higher than the group mean.

#### Theme 1. Stress both drives and undermines adolescents' engagement with mindfulness

Across all three phases (before group, during group sessions, outside of group sessions), stress emerged as a salient topic for adolescents as they reflected on their participation in L2B. There were 3 subthemes within this broader theme.

##### Adolescents enter the program with a sense that mindfulness is valuable for stress management and their experience in the program confirms this beliefgenerating a greater sense of value

Stress served as an initial motivator for adolescents to enroll in the program, particularly as many adolescents entered the program with a preexisting sense of the value of mindfulness as a tool for stress management. One 15-year-old boy (ID #109, High CBE, High AE) said: “Well, my initial thoughts were, that’s gonna probably help me with taking care of my mind and not to have stress take over me.” In some cases, this sense of benefit outweighed initial hesitations. For example, a 12-year-old girl (ID #812, High CBE, High AE) shared, “To be honest, I thought it sounded a little boring at first, but then I thought it could be helpful for managing stress and understanding my feelings a little better.” Although most adolescents did not specify where their beliefs about mindfulness originated, a few mentioned sources such as therapy, school, or family members.

During the program, adolescents’ initial beliefs about the value of mindfulness were reinforced and expanded, as almost all adolescents who were interviewed perceived mindfulness to be an effective strategy for coping with stress. Adolescents cited that mindfulness helped them with general stress; for example, a 12-year-old girl (ID #401, Low CBE, High AE) said: “[Mindfulness activities] helped me feel more relaxed whenever I’m feeling like stressed out or anything.” They also offered concrete examples common in adolescence: “It helped me with like friend drama and like school, like tests and stuff.” (13-year-old girl, ID #427, High CBE, High AE). After the program ended, some adolescents reflected on their experience and concluded that their initial perceptions of mindfulness had been confirmed. A 17-year-old boy shared (ID #615, Low CBE, Low AE): “[Before starting the program] the idea of learning how to cope with that stress in a different way than how I’m used to would be beneficial, I thought at the time. And I definitely think it was.” Others went so far as to say they would recommend the program to others expressly for the stress management benefits: “It’s very helpful, especially during the school year when [teens] get stressed out with like homework or just school in general. So, I would say [teens] should definitely try it.” (15-year-old girl, ID #113, Low CBE, High AE).

##### Stress provides helpful opportunities to develop and practice mindfulness but can also make it harder to engage in mindfulnessboth in and out of group session

Some adolescents noticed that stress was helpful to their developing mindfulness practice, as they were most inclined to apply what they had learned in the program during their most stressful moments. For many adolescents, school-related stressors activated their engagement in independent mindfulness practice, and, in turn, mindfulness increased their sense of self-efficacy to cope with these sources of stress. For example, a 13-year-old boy (ID #425, Low CBE, Low AE) shared: “If I had a test, I was anxious, but I knew that I would be fine because some of the mindfulness practices were going to help me during it.”

Simultaneously, multiple adolescents identified stress as a barrier to fully engaging in the program, particularly by making it more difficult to complete the suggested home practice. Adolescents frequently reported feeling *too* stressed to practice mindfulness. Stress primarily hindered adolescents’ mindfulness practice by either reinforcing a perception that they were too busy or lacked the time, or by negatively affecting their mood and motivation. A 17-year-old girl (ID #512, High CBE, High AE) shared: “Sometimes, with all the stress from school and, like, social stuff, it was hard to find time, or, like, a strong desire to practice it so sometimes I just got caught up in other things. ” Stress also interfered with participants’ desire to attend groups and stay fully present during a mindfulness session. For example, in speaking about an acutely stressful experience that coincided with her participation in L2B, a 15-year-old girl (ID #200, High CBE, High AE) said: “I think that the [session] that was immediately after that [stressful experience], I was less engaged, and my thoughts were definitely wandering.”

Overall, responses reflected that stress could support or hinder adolescent engagement with practice, varying significantly within and across participants and often without a clear explanation. Although there were between-person differences – some adolescents experienced stress as more of a barrier than others – many adolescents described that they experienced both the motivating and discouraging effects of stress on their practice. One 17-year-old girl (ID #512, High CBE, High AE) articulated this contradiction clearly: “Sometimes if I did have a bad day, I was ready to do mindfulness. I kind of had that to look forward to. So, it kind of varied. Some bad days I’d be like, ‘Oh, I don’t want to do this.’ But then other days I’d be like, ‘Oh, I could really use mindfulness right now.’” A 17-year-old boy (ID #615, Low CBE, Low AE) offered a similar insight: “A lot of times you’re not in the mood to practice that sort of stuff because at least for me, I just get frustrated and so stressed. I’m like, I don’t want to deal with this right now. It just feels like another thing. But also, like I said, it definitely gave me a target to kind of use my mindfulness on.”

#### Theme 2: Group dynamics influence engagement for better or worse

Adolescents had much to say about the experience of being in a group, their perceptions of their fellow teenagers, and their preferences related to group learning. Adolescents’ experiences of the group were deeply personal, as well as variable by cohort; however, there were important common threads. There were three subthemes related to group dynamics.

##### Adolescents are attuned to and influenced by others’ engagement levels

Adolescents were highly aware of other group members’ engagement, including others’ level of commitment to and interest in the group, and made assessments about how much they thought others wanted to be there. They were equally attuned to low engagement as well as high engagement. For example, a 13-year-old girl (ID #224, Low CBE, Low AE) noticed boredom and distraction: “A few of them seemed really like interested in the topic, but otherwise some of the other ones seem kind of like bored with [the L2B program].” In contrast, a 14-year-old boy (ID #205, High CBE, High AE) noticed: “Everyone around me seemed very focused, which helped me stay calm” and a 16-year-old participant (ID #513, High CBE, High AE) reflected: “The enthusiasm of the people around me was really helpful. Everyone was really into it. Nobody was like acting like they were bored or disinterested.” These responses highlight that, during sessions, adolescents were interpreting their groupmates’ body language, comments, and behaviors as signals of their engagement and, further, they were influenced by the level of engagement that they perceived.

##### Comfort and commonality with peers foster participation for most adolescents

Adolescents overwhelmingly indicated that they felt comfortable with the group, but they did not feel close to others. The sense that others were non-judgmental was key to fostering feelings of comfort. As described by one 14-year-old boy (ID #205, High CBE, High AE): “No one seemed very judgmental or anything [….] So, I just felt good being around them, like I could be myself.” For some teenagers, a nonjudgemental group setting was more important than feeling close to others. A 12-year-old boy (ID #423, Low CBE, High AE) said: “No one was, like, a really good friends, like, we didn’t know each other, but everyone was just neutral towards each other, like no one was like, judging.” For most teenagers, the sense that others were not judging anyone allowed them to feel comfortable sharing and participating.

Another contributor to adolescents’ sense of comfort in the group was the sense that participants had shared experiences, and this realization supported adolescents’ to openly share and participate. A 17-year-old girl (ID #314, High CBE, Low AE) said: “I felt comfortable because they all shared similarities to me, like when we shared something, there were things that we all had, that we all had similar things.” A 17-year-old boy (ID #615, Low CBE, Low AE) echoed this sentiment saying, “It was easier to be in the class and learn when you know people better and know that they’re going through similar things or struggling with similar issues.” In this way, a sense of commonality supported cognitive-behavioral engagement through facilitating participation as well as affective engagement through fostering comfort and group connectedness.

##### Awkwardness makes interactive learning stressful for some adolescents

For some adolescents, lack of personal closeness in the group setting left them feeling awkward or anxious and reduced their participation. As a 15-year-old girl (ID #200, High CBE, High AE) put it: “I think sometimes it felt a little awkward. We weren’t really warmed up to the people in it and I was a little intimidated to share sometimes.” Similarly, a 12-year-old girl (ID #220, High CBE, Low AE) said: “In the beginning, I was anxious and didn’t want to share as much.” Not knowing other group members well or feeling a lack of social connectedness to peers produced nervousness for some adolescents. A 17-year-old girl (ID #512, High CBE, High AE) spoke specifically about awkwardness, saying, “In the beginning, it was definitely awkwardness with the other kids, and like kind of nervous, definitely to share things, because we were all asked to share how we feel in the moment.” Without feeling a sense of familiarity and connection with others in the group, a subset of teenagers was less inclined to participate actively and fully in the group discussion sections of the L2B program.

#### Theme 3: Adolescents experience, and judge, emotions that they perceive to interfere with engagement 

Adolescents shared extensively on the emotions that they experienced during mindfulness sessions. Most participants experienced feelings of frustration and self-doubt as a result of struggling to train their attention. A 15-year-old girl (ID #113, Low CBE, High AE) said: “I just get frustrated, because it was a little bit hard to always keep my mind on the thing that we were doing. I struggled to not just let my mind wander.” Some adolescents reported that struggling with distraction was unpleasant and they negatively judged themselves for what they perceived as an inability to fully engage in the program. A 17-year-old girl (ID #314, High CBE, Low AE) described: “My least favorite part would probably be getting distracted or thinking about other things that I shouldn’t be focusing on.” When adolescents’ minds wandered, they experienced a sense that they were not participating.

Also, adolescents reported experiencing a sense of calm in group sessions. These peaceful feelings frequently co-existed with the potential for feelings of tiredness or boredom. Just as adolescents perceived that distraction signified suboptimal engagement, they also ascribed negative judgments regarding their feelings of tiredness, lethargy, and boredom. Adolescents described feelings of sleepiness or fatigue as adversely impacting their engagement. These feelings most often occurred during the mindfulness meditation practices. For example, a 15-year-old girl described (ID #113, Low CBE, High AE): “I think that sometimes I was like so calm, I got a little tired during like, a lot of [the practices].” A 13-year-old girl (ID #801, High CBE, High AE) said: “And then I feel like after a while, I was just really relaxed and calm. And then just hearing the talking, I kind of got tired.” Boredom was perceived as an impediment to engagement, particularly during mindfulness meditation practices. A 14-year-old girl (ID #708, High CBE, High AE) described: “I think [feeling bored] made it a little bit more difficult to participate, just because I was kind of distracted and I didn’t really know what was going on around me.” Multiple adolescents described this emotional mix of calm, which they perceived as pleasant, and tiredness or boredom, which they perceived as less pleasant, suggesting mindfulness training invited these emotions to co-mingle and co-occur.

#### Theme 4: Mindfulness is easy to do but hard to practice

Adolescents spoke quite a bit about how easy or effortful mindfulness was for them. The degree to which it felt easy or hard tended to depend on the context of their practice.

##### In session, mindfulness feels easy to do, especially as the weeks go on

Most adolescents shared that they found mindfulness to be easy, which contributed to their sense of calm during group sessions. A 17-year-old boy (ID #615, Low CBE, Low AE) said: “I mean, it wasn’t like you had to do much. There wasn’t a whole ton that you had to do. It was just simple, relaxing, really.” A 12-year-old girl (ID #711, High CBE, High AE) said: “It was never anything like too hard. Like most of it was easy. It was you know very just like um low-key kind of things and like the guided meditations were like really nice.” Teenagers used school as their reference point for effort, describing that L2B felt far more easeful, even passive, by comparison. Compared to school, which could feel “tedious or hard to do,” in the L2B program, “you just went there, you were there for like an hour-ish, you like learned some things about how you might use [mindfulness] to help yourself and then you just like left” (13-year-old girl, ID #818, Low CBE, Low AE). This sense of ease was something that teenagers appreciated about the program. In addition, adolescents reported that feeling calm and at ease supported their participation. For example, an 18-year-old boy (ID #514, High CBE, High AE) said, “Being calm made it easier to share.” In-session activities required minimal effort for many adolescents, which facilitated a sense of ease and, in turn, made them more inclined to participate.

Despite most adolescents emphasizing that mindfulness was simple, easy, and involved minimal effort, some teenagers described some initial barriers to understanding the concept of mindfulness, a key dimension of cognitive-behavioral engagement. As a 17-year-old boy (ID #615, Low CBE, Low AE) put it: “Because [mindfulness] is such a unique thing to do, it was kind of hard at first, at least to kind of grasp the main idea for a minute. And then once I kind of felt my emotions more, it definitely seemed to click. But like getting used to relaxing and thinking about breathing and thinking about how you feel, that was definitely kind of a learning curve.” Another younger, 12-year-old girl (ID #401, Low CBE, High AE) cited similar difficulties with understanding: “Some things were kind of confusing, but then, like, whenever we talked more about it, it got more like understanding for me. So like at the end, I understood it, but at first I didn’t really understand it much.” For at least some of the adolescents we interviewed, cognitive-behavioral engagement was initially limited by their difficulty understanding the concept but was ultimately supported through multiple opportunities to discuss mindfulness and practice.

##### Home practice is hard, but it helps to approach it like building a habit

Despite teenagers’ sense of ease of practicing mindfulness during L2B sessions, the majority had difficulty establishing a home mindfulness practice. Most teenagers viewed home practice as important. For example, a 17-year-old boy (ID #615, Low CBE, Low AE) said: “It was the at-home exercises that really helped to cement the ideas [from sessions].” Yet, teenagers frequently struggled with remembering to practice. As a 13-year-old girl (ID #801, High CBE, High AE) noted: “Sometimes I would just forget to do it. Just like, I just need to remember to do it.” School, extracurriculars, social activities, and generally having a busy schedule contributed to their tendency to forget. However, when teenagers were able to approach mindfulness practice as building a habit, through using self-reminders or practicing at specific times of the day, they had more success with sustaining a regular home practice. A 14-year-old girl shared (ID #708, High CBE, High AE): “They gave us, like, mindfulness dots, like, you take three deep breaths whenever you see it. I use that a lot in my life. I mean, I have one, like, on my water bottle, on my mirror in my room, and I’ve noticed myself really remembering to do that and use those, and that’s sort of a habit that I’ve formed.” These frequent visual reminders on everyday objects helped many adolescents build mindfulness into daily routines.

##### Adolescents choose practices that are accessible and helpful

When teens practiced mindfulness outside of sessions, they did so in a few specific ways. Breathing practices were overwhelmingly the most cited home practice activity. Participants felt that conscious breathing had a measurable impact on their stress, state of mind, and mood. In many cases, they talked about controlling the breath or breathing in a particular way (e.g., taking deep breaths). A 14-year-old girl (ID #708, High CBE, High AE) valued this practice, saying: “I struggle with like anxiety sometimes, and it’s important for me to know like how I can control my breath.” For the majority of teens, their home breathing practice involved deepening or slowing the breath, which aligns with part of the opening practice taught during each session of L2B. Adolescents did not discuss independently practicing the exercise of noticing and allowing the breath to be as it is, which is another breath awareness practice taught in L2B.

Related to breathing, which for many youth was synonymous with “taking a pause,” teenagers appreciated and tried to integrate the practice of slowing down into their daily lives. One 12-year-old girl (ID #401, Low CBE, High AE) shared: “Now I kind of walk a little slower to like, enjoy things around me whenever I walk.” Teenagers perceived that they became better able to interrupt habitual patterns of behavior with a mindful pause. A 15-year old girl (ID #813, High CBE, High AE) shared how she changed her phone use with this practice of pausing in combination with the visual assistance of mindful dots: “We’ve got the little dot circles and I put one on my phone, and it just made me take a minute and just be mindful instead of like just going on my phone or like going to text someone, just being mindful about that.”

Adolescents also incorporated more informal types of mindfulness practice into their day-to-day lives through practicing awareness of their bodily sensations and surroundings. For example, in describing their home practice, a 16-year-old participant (ID #513, High CBE, High AE) said: “I usually, like, took a breath, thought about, like, how my body feels, and like, focus on, like, the physical sensation of it, and it was, it was really nice, because it like, really grounded me.” This present-moment awareness extended to more unpleasant sensations as well. For example, a 13-year-old girl (ID #801, High CBE, High AE) shared: “In tense situations, I probably like just feel my stomach because that gets really tense and like I, my stomach flexes. And so, then once I take a second to realize that my stomach is like flexing and super tense, then I’m like, okay, I need to like calm down.” Adolescents also enhanced their awareness beyond their internal experience to what was going on around them, and they commonly described that they began noticing new details about everyday experiences. A 12-year-old girl (ID #401, Low CBE, High AE) said: “For the home practices I did, like walking like in the hallways, mindfully, and I feel like that helped me, because, like it made me realize that there was more things than like, I thought there was going on in the halls.” Teenagers expressed the unfolding of mindfulness in their daily lives as an interesting and eye-opening process. A 12-year-old boy (ID #423 Low CBE, High AE) said: “It’s a little interesting, like, how you can like, notice more things about the world and stuff.” Outside of group sessions, teenagers chose to anchor their attention in internal sensations and emotional experiences and/or external stimuli, and they appeared to find that this way of approaching daily life experiences was an accessible but powerful way to practice what they had learned in sessions.

### Mixed Methods Results

We merged quantitative and qualitative data by examining qualitative themes by level of engagement and developing meta-inferences. These results are reflected in a joint display (Table 3). In general, qualitative themes were applicable to and relevant for adolescents of all engagement levels, although there was some variability by engagement level in the specifics of how adolescents articulated and endorsed those themes.

**Table 3:** Joint Display Merging Quantitative and Qualitative Results: Thematic Results by Quantitative Engagement Level

Theme	Low Engagement^1^ (*n* = 6)	Mixed Engagement^2^ (*n* = 5)	High Engagement^3^ (*n* = 14)	Meta-inference
Theme 1: Stress both drives and undermines adolescents’ engagement with mindfulness	“If you’re like really busy doing something, you can’t just stop what you’re doing to think about being mindful. ” (#224, Low CBE, Low AE) ‬‬‬‬‬‬‬‬‬ “If I had a test, I was anxious, but I knew that I would be fine because some of the mindfulness practices were going to help me during it.” (#425, Low CBE, Low AE)	“Whenever I got stressed out and didn’t really know what to do, I took a break away from it and just started breathing.” (#401, High CBE, Low AE)	“I still have the dots from mindfulness and I definitely still use them a lot and find them as like a really good way to calm down if you’re having a chaotic day ” (#711, High CBE, High AE) ‬‬‬‬‬‬ “On top of other stuff that I have going on in my life, like school and other things, it was kind of difficult to retain the information and practice.” (#708, High CBE, High AE)	Adolescents’ ability to apply mindfulness in moments of stress did not appear to vary by engagement level. Adolescents across all engagement levels were, at times, able to bring mindfulness skills to stressful moments and, at other times, unable to utilize mindfulness under stress.
Theme 2: Group dynamics influence engagement for better or worse	“I don’t really like being in like big groups of people. So when I first joined the group, I was really nervous. ” (#224, Low CBE, Low AE) ‬‬‬	“I didn’t feel super close to anyone, but, like, I knew everyone’s name.” (#113, low CBE, high AE).	“I definitely think I’m just a very outgoing person, so that led me to share more, but also just the setting of it. It was very calm, it was very chill. So I just felt like it was okay to share.” (#514, High CBE, High AE) ‬‬‬	Engagement may be confounded with personality traits, such that adolescents who tended to report higher engagement also tended to enjoy the group learning setting, whereas adolescents with lower engagement tended to be less comfortable in a group.
Theme 3: Adolescents experience and judge, emotions they perceive to interfere with engagement	*Not represented*	“I wasn’t doing good, or what I was supposed to be doing, because I was bored. (#314, High CBE, Low AE) ‬‬‬ “During longer mindfulness practice, in the beginning, I was really engaged, but as the time went by longer, I felt myself engaging in it a lot less, because I kind of got bored.” (#401, High CBE, Low AE)	“When we were doing a really lengthy breathing exercise, I guess I kind of got bored and distracted, and that was a bit difficult.” (#708, High CBE, High AE) “Sometimes I felt a little uncomfortable [and] I think it affected my participation because when I would be like that my imagination would kick in and I would just let my mind wander, and I wouldn’t participate.” (#205, High CBE, High AE)	Although adolescents of all engagement levels experienced boredom at times, only adolescents with mixed and high engagement reflected judgment of the ways that boredom or discomfort impacted their engagement.
Theme 4: Mindfulness is easy to do, but hard to practice.	“It was easy and you can do it any time really. And it’s so simple that it can work in any situation, really.” (#615, Low CBE, Low AE) “I’m a very forgetful person, so sometimes just simple things like forgetting.” (#815, Low CBE, Low AE)	“Trying to change my routine, I just like can’t do that very well.” (#220, High CBE, Low AE) “I think sometimes I would just forget, like I would just be in my head and I would forget to [practice at home].” (#113, Low CBE, High AE)	“After a few weeks of doing it, I got in some of the habits, I got better sleep, putting away my phone and doing that practice right before I went to bed.” (#205, High CBE, High AE) “I would always forget to do the mindfulness practices at home.” (#218, High CBE, High AE) “It was never anything too hard, like most of it was easy.” (#711, High CBE, High AE)	Forgetting to practice affects all adolescents, regardless of engagement level. Higher engaged adolescents consciously created habits to help them remember to incorporate regular mindfulness practice.

^1^Low Engagement: Adolescents who reported engagement scores lower than the group mean on both dimensions. ^2^Mixed engagement: Adolescents who reported engagement scores lower than the group mean on one dimension and higher than the group mean on another dimension. ^3^High engagement: Adolescents who reported engagement scores higher than the group mean on both dimensions

## Discussion

In this mixed methods study, we used quantitative data to characterize the level of multidimensional engagement among adolescents receiving an MBI, and to purposively sample across the spectrum of engagement a subset of adolescents to participate in post-program interviews. We then merged quantitative and qualitative results to examine thematic results by quantitative engagement and develop meta-inferences. Engagement was generally high, with the cognitive-behavioral dimension of engagement significantly higher than affective engagement. As intended, we succeeded in recruiting an interview sample that reflected a range of engagement levels. This variability was reflected in the nuanced and, in some cases, dialectical themes that were generated in qualitative analysis. We found that adolescents generally endorsed similar experiences of engagement in the MBI regardless of their quantitative self-report engagement scores – as reflected by minimal variability in themes by engagement level – which may suggest a need for measures of engagement specific to MBI that offer more precision and sensitivity/specificity. Overall, adolescents’ perspectives on engagement in MBI reflected areas of alignment with prior conceptualizations of engagement and highlighted several distinctive characteristics of engagement in this specific context. Our analyses of adolescents’ responses suggest that there are important pathways by which engagement is linked to other MBI mechanisms and outcomes, including degrees of stress and the consistency of home mindfulness practice. From a pragmatic perspective, although descriptive statistics show that self-reported engagement was high, thematic results indicate key opportunities before, during, and outside of MBI sessions to further support adolescent engagement.

Engagement scores were uniformly high, particularly for cognitive-behavioral engagement, which warrants careful interpretation. One possibility is the presence of ceiling effects, which may have reduced the ability of the measure to distinguish between levels of engagement. This pattern could be attributable to social desirability bias, particularly given that surveys were completed immediately following sessions in the presence of facilitators. Though this data collection strategy may have minimized recall bias and supported the collection of more complete data by providing dedicated time for adolescents to complete surveys, it may also have increased the likelihood of socially desirable responding or perceived expectations to report higher engagement. Notably, high quantitative engagement scores contrast with qualitative reports of boredom, difficulty sustaining attention, and variable levels of participation. This discrepancy suggests that traditional quantitative self-report measures of engagement may capture compliance or participation more readily than adolescents’ internal experiences of engagement, which are better captured by qualitative reports. The more varied qualitative responses may also reflect the use of interviewers who were independent of intervention delivery (as opposed to quantitative data, which was collected by facilitators). Taken together, these findings highlight the importance of integrating multiple methods to more comprehensively assess engagement and suggest that refinement of engagement measurement in the MBI context may be warranted. Specifically, qualitative insights suggest the need to refine measurement such that it is possible to assess internal attentional engagement – potentially through momentary assessments or physiological measures (Siennicka et al., 2019), as well as capture co-occurring emotional states (e.g., calm alongside boredom) and incorporate social-contextual influences on engagement that were not assessed in the current measures.

Adolescents primarily viewed mindfulness as a tool that they could use for reducing stress (Themes 1.1 and 4.3). This sense of the usefulness of mindfulness was an important driver of both initial enrollment and sustained engagement throughout the program, including home practice completion and involvement during MBI group sessions. This finding is consistent with evidence that having a higher sense of value for mindfulness prior to starting an MBI predicts higher participation (in the form of attendance) in MBI (masked for review). Our findings expand upon this prior evidence by suggesting that the relationship between sense of value and engagement may not simply be unidirectional, but rather resemble an upward spiral. Adolescents described that their sense of value supported their engagement which, in turn, generated an even greater sense of value for mindfulness. More research is needed to directly test the possibility of an upward spiral phenomenon and quantify the magnitude of these associations. Targeted efforts to support adolescents’ sense of value before and throughout MBI may be helpful in promoting multiple forms of engagement. For instance, it may be helpful to gather information at the outset of MBI about each adolescent’s individualized reasons for participating. Facilitators could then amplify that sense of value through the examples they provide during MBI activities and through tailoring suggested home practices. For example, if adolescents share that they hope mindfulness will help them sleep better, facilitators could share suggestions for home practice that are aligned with that priority (e.g., nighttime body scan).

Mindfulness, however, is not simply a tool for stress reduction, and, theoretically, adopting a strictly instrumental approach to mindfulness may reduce its benefits (Shapiro et al., 2018). Thus, although our data suggest that sense of usefulness supports certain forms of cognitive-behavioral engagement such as participation, it may limit other forms of cognitive-behavioral engagement like understanding of the complex construct that is “mindfulness.” Indeed, despite generally high quantitative scores on cognitive-behavioral engagement (of which an aspect is understanding), interview data reflected an underdeveloped understanding of some of the key foundational attitudes posited to be central to MBI benefits, including nonjudgment, nonstriving, acceptance, and letting be (Kabat-Zinn, 1990). These aspects of mindfulness are theorized to be particularly important for mental health outcomes (Lindsay & Creswell, 2017, 2019). These qualities are underscored throughout the L2B curriculum, including a dedicated session on self-compassion and nonjudgment, a practice focused on noticing and accepting unpleasant emotions, and emphasis throughout L2B that there is no “right or wrong” way to practice mindfulness. However, adolescents in this study judged themselves critically for experiencing boredom and tiredness during activities and practices, and experienced frustration with what they perceived was slow progress in developing mindful attention. This tendency to judge affective experience could have contributed to the significantly lower quantitative scores on affective engagement, relative to cognitive-behavioral engagement. It is common for novice practitioners of mindfulness to engage in effortful striving, or making a self-improvement project out of mindfulness, and to struggle with self-judgment of their progress (Kabat-Zinn, 1990; Lutz et al., 2015). Adolescents may be even more susceptible to this attitude toward mindfulness, given the highly achievement-oriented cultures of most school settings where US adolescents spend a large portion of their time (Shannon, 2024). Or, these results may reflect that, rather than a lack of understanding, adolescents are struggling to apply these challenging concepts to their own experiences, which is not surprising developmentally because the capacities for this type of cognitive flexibility are still developing (Gopnik et al., 2017). In either or both cases, adolescents may need more reinforcement of mindfulness beyond a stress-management tool, likely through a combination of increased content (to support deeper understanding) around nonjudgment, nonstriving, and acceptance, as well as increased practice opportunities and a focus on what these attitudes look like in “real life” (to support skill development). A program consisting of six 60-minute sessions provides relatively little exposure to these challenging concepts, although this dosage is in line with common practice in the field (Felver et al., 2024; Kuyken et al., 2022). Future research should examine trajectories of understanding and skill acquisition of these core components of mindfulness to identify whether additional sessions or tools to reinforce concepts related to the more complex facets of mindfulness are merited.

Our results lend support for conceptualizations of affective engagement that include emotional response to elements of the social context (e.g., fellow learners, facilitators) (Fredricks & McColskey, 2012; Wong & Liem, 2022). Although L2B was developed for school-based delivery (Broderick, 2021; Marshall et al., 2024), in this study, we delivered L2B outside of school. Participants did not know one another, which shaped adolescents’ affective engagement – some felt at ease and trusting, others felt anxious, and none felt particularly close (Themes 2.2 and 2.23). These affective responses to the group seemed to shape cognitive-behavioral engagement – specifically participation – to some degree (Theme 2.2). That is, adolescents who did not feel comfortable expressed that they participated less because of this discomfort. The centrality of the group dynamic, and in particular findings related to adolescents’ keen attunement to others’ behavior (Theme 2.1), is consistent with research showing that adolescence is a period of increased social interest, evaluation, and comparison (Crone et al., 2022). On one hand, closeness to group members has not been posited as an essential ingredient to experiencing the benefits of group mindfulness training (Felver et al., 2022), and our findings align with this perspective for the most part, though there are individual differences in the degree to which closeness is valued. On the other hand, greater group closeness or social cohesion may encourage more participation and positive affect during sessions (Hinsz & Bui, 2023). When delivering MBI in out-of-school contexts, modifications to the program content or structure may be needed to foster greater connection between participants. For example, mixed-age groups, such as those in the present study, may influence group dynamics and engagement, although this was not directly examined. Participatory, collaborative research conducted with adolescents to inform these refinements is expected to be especially fruitful (Freire et al., 2022). Importantly, the instrument we used to quantitatively assess affective engagement did not contain items related to adolescents’ response to elements of the context; rather, questions were limited to adolescents’ own internal emotional experience. Our qualitative results suggest that future work may be needed to refine instruments designed to measure affective engagement in MBI so that they capture social contextual influences.

Another key finding related to affective engagement was that adolescents perceived that certain emotions reduced their cognitive-behavioral engagement, with important MBI-specific implications. In the context of MBI, affective engagement takes on an additional layer of importance because central to mindfulness practice is noticing and accepting the entire range of emotional experiences. In future research, it may be important to distinguish between participants’ affective engagement during mindfulness *learning activities* versus mindfulness *practices*, as affective engagement may play distinct roles during each of these intervention elements. For example, boredom during learning activities may interfere with positive outcomes, as we could imagine that it might lead a participant to “check out,” miss key information that could help them in the process of learning mindfulness, or even opt out entirely of trying to practice. However, boredom experienced during mindfulness practice might be important for increasing distress tolerance (i.e., the perceived or actual capacity to withstand discomfort) by supporting greater openness to and acceptance of unpleasant experiences and/or reducing negative thought patterns around the distress (e.g., rumination, anxiety) (Brown et al., 2007). Indeed, cultivation of feelings of boredom or restlessness is a purposeful component of experiential mindfulness practice (e.g., in the L2B session dedicated to practicing mindfulness of emotions). Even brief mindfulness training can increase distress tolerance in adolescents (Carpenter et al., 2019; Liu et al., 2013). Thus, relatively low-intensity unpleasant emotional states like boredom that are likely to arise during mindfulness practice theoretically facilitate benefits. However, adolescents in this study were not fully able to apply mindful nonjudgment (i.e., self-compassion) to affective experiences of boredom, frustration, tiredness, and others; rather, they considered them as barriers to engagement instead of natural and expected, constantly changing, emotional experiences. Interestingly, the most engaged adolescents tended to report a greater tendency to judge these types of emotions. Thus, ensuring that engaged participants balance their effort with mindful nonjudgment may reflect a unique opportunity and challenge for mindfulness instruction, relative to other contexts in which judgment of one’s efforts is less problematic. More research is needed on the role of pleasant, neutral, and unpleasant emotions in the context of adolescent MBI, in both the didactic/discussion activities and the mindfulness meditation practices themselves.

Many adolescents were emphatic about how easy mindfulness felt in session (Theme 4). This finding raises important questions about the role of effort in operationalizing MBI engagement, particularly given that cognitive-behavioral engagement is defined as “effortful participation.” It highlights potential limitations of traditional engagement frameworks for the MBI context, where effective engagement may not always align with effortful or outwardly observable participation. This qualitative theme also helped explain quantitative patterns, particularly the higher levels of cognitive-behavioral relative to affective engagement. Adolescents frequently described observable participation (e.g., attending, contributing, completing activities) as straightforward, while simultaneously reporting mixed or negative emotional experiences such as boredom, frustration, or self-doubt. It is also possible that when adolescents said easy, they may have really meant accessible or basic; mindfulness is often said to be simple, but not easy (Goldstein, 2007). Adolescents’ accounts of trying to practice mindfulness in moments of stress (Theme 1.2), emotional experiences of frustration and distraction during practice (Theme 3), and the diligence needed to establish home practice (Theme 4.2) suggest that they did find aspects of learning and practicing mindfulness more challenging. Nevertheless, the role of effort in MBI is likely more nuanced than other learning contexts. Mindfulness practice does require attention, willingness, and persistence in the face of distraction, but the effortful striving of classroom learning is not relevant and, when the impulse toward that form of striving arises, it can be noticed and allowed. Interviews with adolescents that are focused on understanding the unique form of effort that is most conducive to MBI would be valuable. It may also be worthwhile to interview adolescents immediately after they do a mindfulness practice, to ask them about their experience of effort and striving while doing so, to reduce inherent retrospective biases in reporting about past experiences.

In contrast with practices in-session, which felt easy to adolescents, home practice was perceived as challenging (Theme 3.1), which aligns with evidence that rates of home mindfulness practice are low for adolescents (Kuyken et al., 2022; Montero-Marin et al., 2022; Quach et al., 2017). However, adolescents said home practice was made easier by using external cues and repetition, and higher engaged adolescents made better use of these types of habit-building strategies. Although L2B integrates several evidence-based strategies for habit change (e.g., planning, providing a salient cue, continued monitoring and positive feedback; Lally & Gardner, 2013), our findings suggest that adolescent home practice could be bolstered by additional supports designed to encourage the habit of mindfulness and discourage the habit of mindlessness. There may be opportunities in future research for adaptive intervention elements, whereby habit-forming strategies, such as the use of environmental cues and behavioral tracking, are targeted to lower engaged adolescents, since these adolescents seemed less likely to make use of such strategies.

Stress was both a barrier and facilitator of home practice for adolescents of all engagement levels (Theme 1.2). On one hand, stress undermined home practice engagement. If we consider that, prior to L2B, adolescents would likely be in the habit of *not* practicing mindfulness when stressed, the tendency to opt out of practice in moments of stress aligns with experimental research showing that stress is associated with greater habitual behavior and reduced goal-directed behavior (Schwabe & Wolf, 2010; Smeets et al., 2019). On the other hand, stress sometimes prompted and promoted home practice. Increased mindfulness can reduce avoidant coping in the face of stress (Weinstein et al., 2009); thus, it is possible that in these moments, adolescents had higher levels of state mindfulness and, in turn, were more likely to engage in meeting with stressors with more awareness, nonjudgment, and ease or balance. Future research utilizing ecological momentary assessment is well-suited to probe these different patterns of the effect of stress on engagement in home practice.

### Limitations

This study is limited by our sample, as the data were limited to the perspectives of adolescents who volunteered to participate in an MBI. Adolescents participating in school-based MBI, for example, may have a different experience of engaging in this type of intervention, particularly if participation is required rather than optional. Our sample was also majority non-Hispanic White, and all adolescents lived in the same mid-sized city in the Western US. Additionally, girls were overrepresented in the interview sample. Given documented gender differences in emotional expression (O’Kearney & Dadds, 2004) and responsiveness to MBI (Kang et al., 2018; Lassander et al., 2021), this imbalance may have influenced qualitative findings. Interviews were retrospective and thus are subject to recall bias. However, we made efforts to limit the amount of time that passed between the end of the intervention and the interview (2–14 days). Our sample included a wide age range, and the same interview guide was used for all participants, which may have been a limitation. The interview guide was designed to be developmentally appropriate; however, future research could benefit from participatory methods that include adolescents on the research team to ensure that the guide is developmentally appropriate.

## Conclusions

This study sheds light on the subjective experience of adolescents participating in MBI with a focus on multidimensional engagement. Adolescent responses suggested that engagement is an important process for MBI efficacy. Our results also highlight the ways in which engagement in the MBI context is distinctive, relative to other contexts where engagement has been more rigorously studied. In particular, an MBI-specific conceptualization of engagement likely requires that we redefine the role of effort in cognitive-behavioral engagement and the role of unpleasant emotions in affective engagement. Additionally, adolescent responses indicated that external factors influence engagement, and there are likely opportunities before, during, and outside of MBI sessions to support engagement. With more context-specific, intentionally designed measures of engagement and robust study designs that allow researchers to experimentally test influences on engagement, it may be possible to better understand and, ultimately, optimize MBI to be maximally engaging and effective for supporting adolescent mental health outcomes.

## Data Availability

Data are available by request from the corresponding author.
